# STAT3 Inhibition in a Murine Model of Human Breast Cancer-Induced Bone Pain Delays Onset of Nociception

**DOI:** 10.1080/24740527.2019.1641060

**Published:** 2019-07-30

**Authors:** Manu Sharma, Katja Linher-Melville, Jesse Sidhu, Peter Nakhla, Gurmit Singh

**Affiliations:** Department of Pathology and Molecular Medicine, McMaster University, Hamilton, Ontario, Canada

**Keywords:** STAT3, cancer-induced bone pain, nociceptive, allodynia, breast cancer, cancer, cancer pain

## Abstract

**Introduction/Aim**: Alterations in extracellular glutamate levels have been previously found to contribute to cancer-induced bone pain (CIBP). Increased activity of system xc-, a cystine-glutamate membrane antiporter, has been previously implicated in our lab in these nociceptive behaviours. System xc- subunit xCT, is further positively regulated by signal transducer and activator of transcription 3 (STAT3). In the current investigation, we hypothesized that DR-1–55-mediated inhibition of pSTAT3 will lead to decreased nociceptive behaviours in a validated xCT overexpression model of CIBP.

**Methods**: Using a murine xenograft CIBP model, a high glutamate-releasing xCT/pSTAT3 overexpressing human breast cancer cell line (T47D clone) was injected into the distal epiphysis of the femur of female nude mice. Nociceptive behaviours were monitored through automated von Frey, dynamic weight bearing, and open field testing for the study duration. Three weeks after cell inoculation, a 14-day schedule of intraperitoneal injections of DR-1–55 or vehicle were administered.

**Results**: In vitro, there were elevated levels of glutamate, IL-6, and IL- 1β exhibited in the T47D clone. These cells also showed significant nociceptive behaviours earlier than the T47D wild type (WT). Treatment with DR-1–55 significantly delayed the onset and severity of spontaneous and induced nociceptive behaviours, as seen with behavioural testing.

**Discussion/Conclusions**: This study shows that targeting pSTAT3 may be a viable treatment when managing CIBP, and can be a molecule of interest in future studies.

