# Supplementary material for: STAT3 Inhibition in a Murine Model of Human Breast Cancer-Induced Bone Pain Delays Onset of Nociception
Source: Can J Pain. 2019 Jul 30;3(2):69. doi: 10.1080/24740527.2019.1641060 (PMC8730670; doi:10.1080/24740527.2019.1641060)
Supplement: Supplemental Material [file UCJP_A_1641060_SM3214.pdf]

STAT3 Inhibition in a Murine Model of Human Breast Cancer-Induced Bone Pain Delays Onset of Nociception

Manu Sharma, Katja Linher-Melville, Jesse Sidhu, Peter Nakhla and Gurmit Singh

Department of Pathology & Molecular Medicine, McMaster University, Hamilton, Canada

Introduction

Signal Transducer and Activator of Transcription (STAT) proteins are involved in cancer inflammation[1], redox homeostasis[2], and XCT regulation[3,4]. Phospho-STAT3 (pSTAT3), is constitutively active in triple negative human MDA-MB-231 cells, which release high levels of glutamate through system xc-. We have previously characterized a T47D XCT, pSTAT3 overexpression cell line[4] to evaluate the *in-vivo* effect of DR-1-55 on bone cancer induced nociceptive behaviour.

We hypothesized that DR-1-55-mediated inhibition of pSTAT3 will lead to decreased nociceptive behaviours in a validated XCT overexpression model of cancer-induced bone pain (CIBP)

Methods

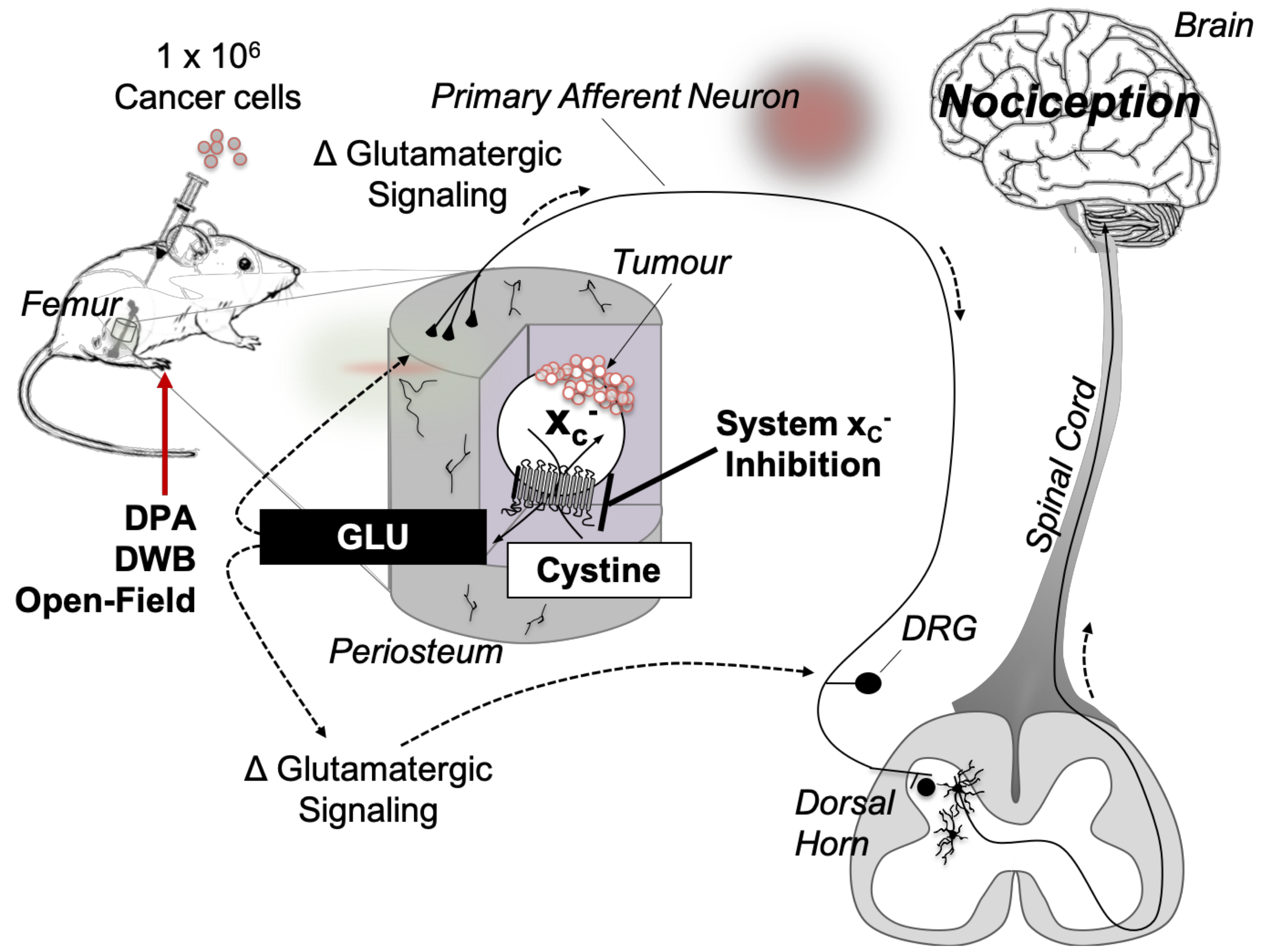

Results

Glutamate Release

Fig 1. Glutamate (A), IL-6 (B), and IL-1β (C) release compared to WT for SH-4-54 resistant clones in MDA-MB-231 and T47D cells.

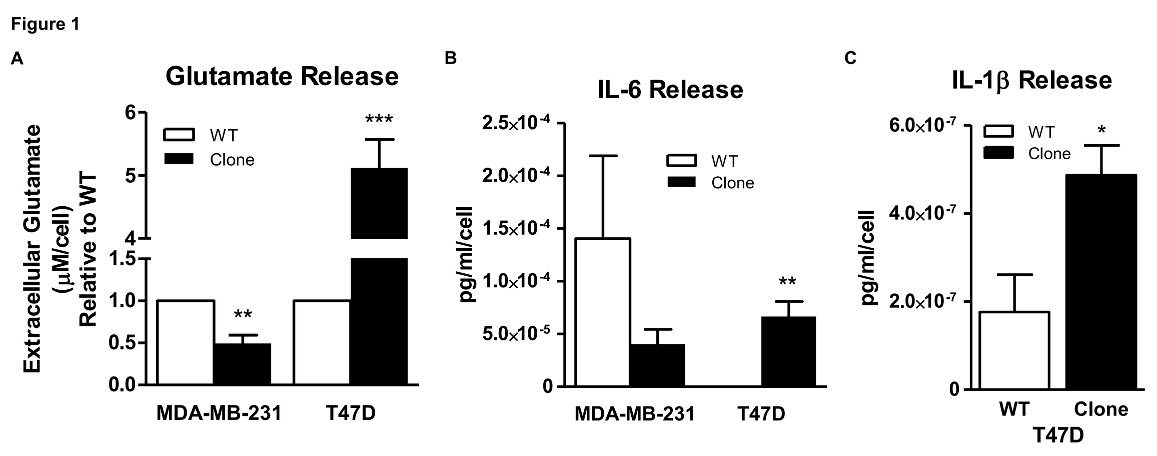

Results

Subcutaneous Growth of T47D cells

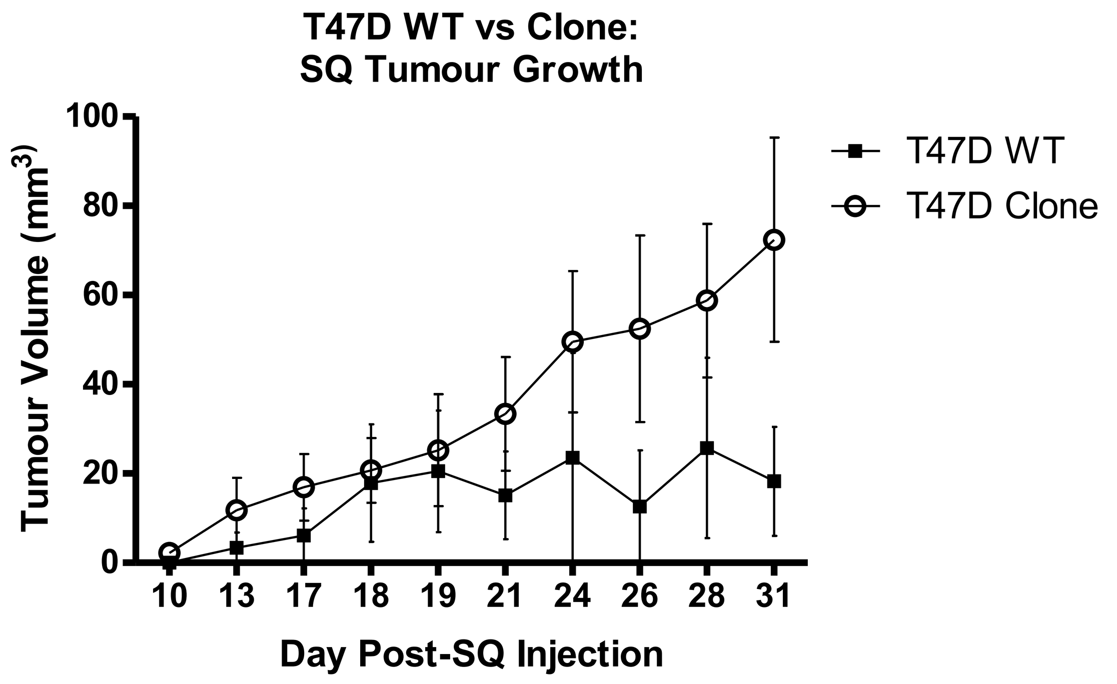

Fig 2. Tumour volume over the course of 31 days in mice injected with subcutaneous T47D WT (n=4) and T47D SH-4-54 resistant clones (n=4). Results are not statistically significant.

Validation of the Cancer-Induced Bone Pain Model

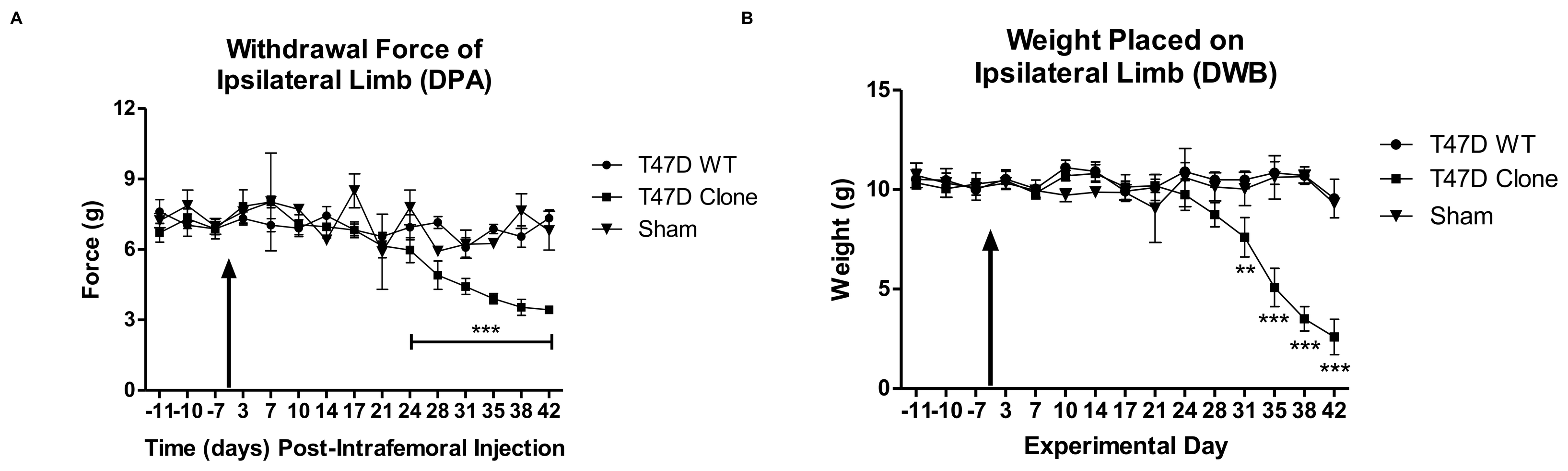

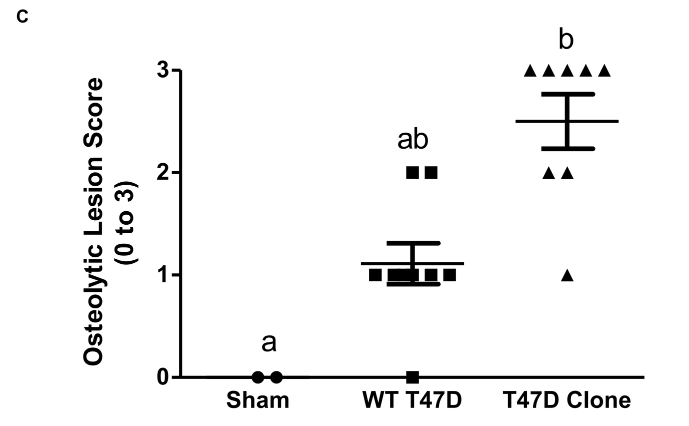

Fig 3. A) Comparison of the withdrawal force between the 3 groups, with significant differences seen past day 24. **B)** Weight placed on ipsilateral hind limb between the 3 groups. Results were significant past day 31. **C)** Analysis of radiographic data highlighting the level of osteolytic lesions in the 3 groups. Results were not statistically significant.

Treatment of T47D SH-4-54 resistant clones with DR-1-55

Fig 4. Mice were inoculated at day 0, and DR-1-55 treatment began on day 21. At day 29, a significant difference was seen between withdrawal force of the treatment group and the vehicle group.

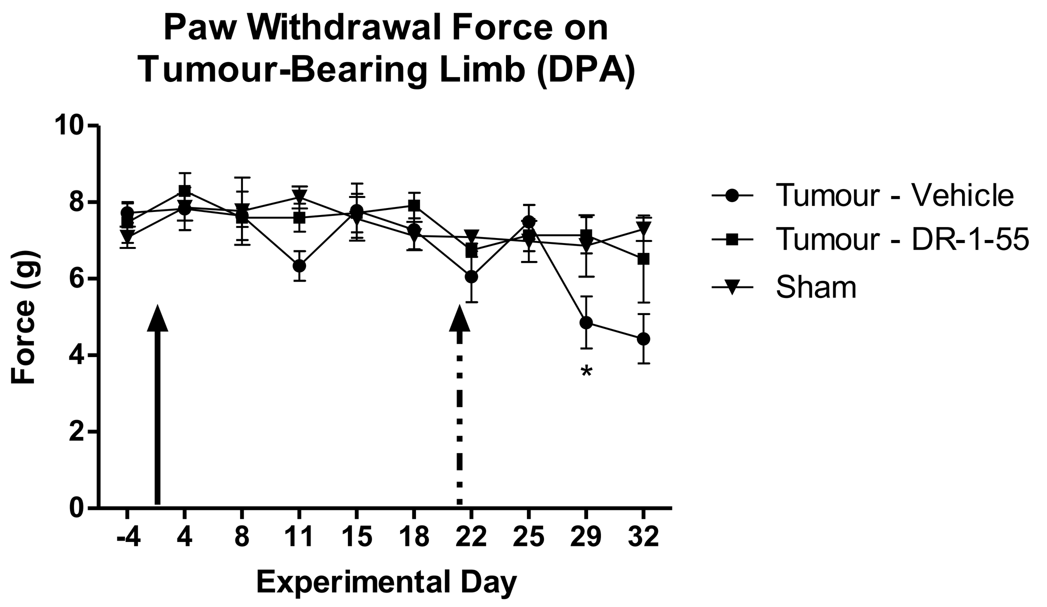

Results

Fig 5. This data shows that mice with vehicle treatment place less weight on the tumour-bearing leg than mice receiving DR-1-55 treatment.

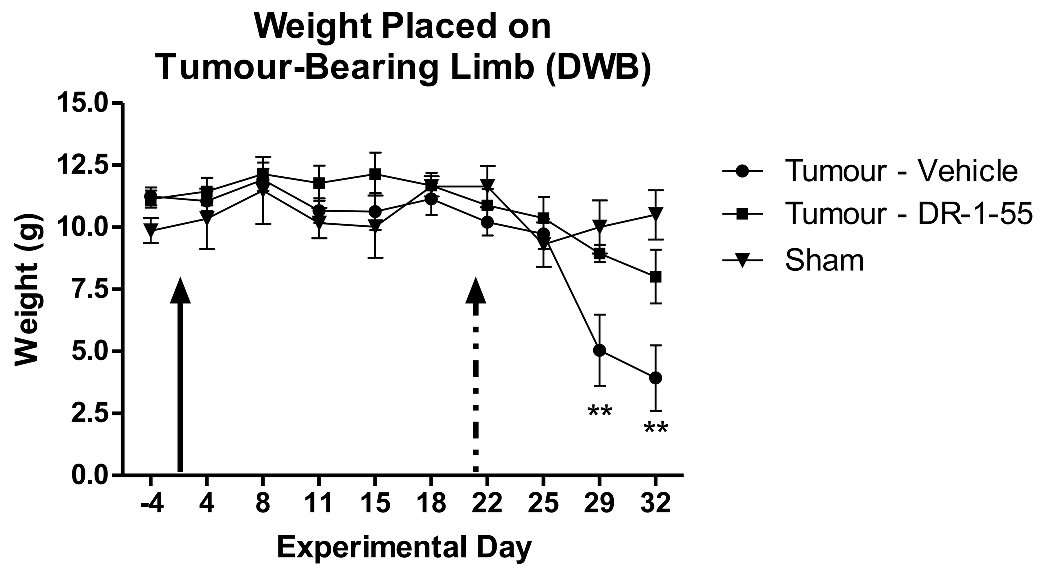

Fig 6. The visual assessment of nociception shows increased nociceptive behaviours in mice in vehicle group rather than treatment group. Results are significant past Day 29.

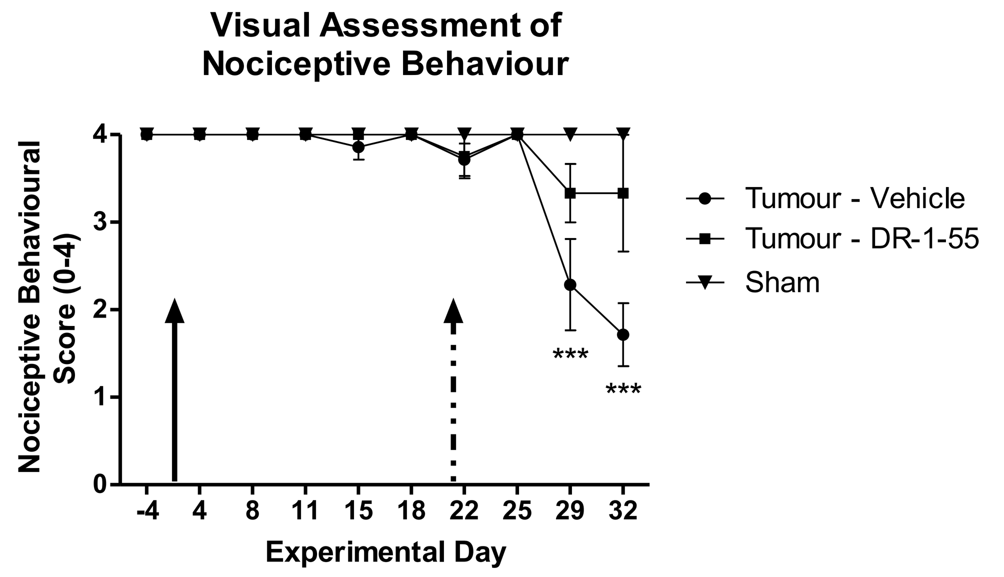

Conclusions

- T47D SH-4-54-resistant clones release significantly greater glutamate, Il-6, and IL-1β than WT
- While T47D clones trend towards a greater subcutaneous tumour size, results are not significant
- T47D clones showed significant differences in behavioural tests compared to WT, in both DPA and DWB, but not osteolytic lesion scoring.
- DR-1-55-treated T47D SH-4-54 resistant clones showed delayed nociceptive behaviours in DWB, DPA, and open-field testing compared to WT

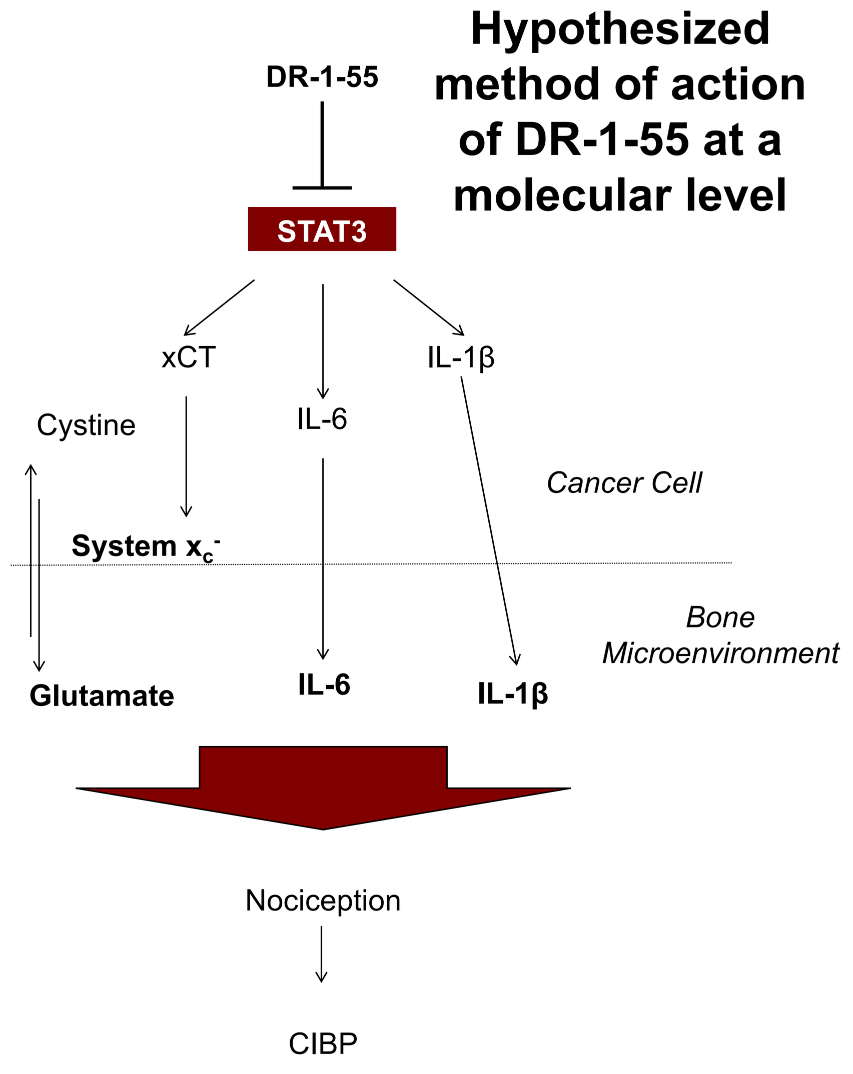

Acknowledgements

We wish to thank the Michael G. DeGroote Institute for Pain Research and Care, the Canadian Institutes of Health Research, and the Canadian Breast Cancer Foundation for supporting this project.

1. Yu H, Pardoll D, Jove R. STATs in cancer inflammation and immunity: a leading role for STAT3. Nature reviews Cancer. 2009;9(11):798-809. Epub 2009/10/24.
2. Linher-Melville K, Singh G. The complex roles of STAT3 and STAT5 in maintaining redox balance: Lessons from STAT-mediated xCT expression in cancer cells. Molecular and cellular endocrinology. 2017. Epub 2017/02/17.
3. Linher-Melville K, Haftchenary S, Gunning P, Singh G. Signal transducer and activator of transcription 3 and 5 regulate system Xc- and redox balance in human breast cancer cells. Molecular and cellular biochemistry. 2015. Epub 2015/04/22.
4. Linher-Melville K, Nashed MG, Ungard RG, Haftchenary S, Rosa DA, Gunning PT, et al. Chronic Inhibition of STAT3/STAT5 in Treatment-Resistant Human Breast Cancer Cell Subtypes: Convergence on the ROS/SUMO Pathway and Its Effects on xCT Expression and System xc- Activity. PloS one. 2016;11(8):e0161202. Epub 2016/08/12.
